# A Potential Partnership between Genetics and the Oral Microbiome in Children Displaying Periodic Fever/Aphthosis/Pharyngitis/Adenitis Syndrome

**DOI:** 10.3390/ijms242115505

**Published:** 2023-10-24

**Authors:** Donato Rigante, Lea Calò, Alessandro Ciavarro, Jacopo Galli

**Affiliations:** 1 Department of Life Sciences and Public Health, Fondazione Policlinico Universitario A. Gemelli IRCCS, Largo A. Gemelli 8, 00168 Rome, Italy; 2 School of Medicine, Università Cattolica del Sacro Cuore, 00168 Rome, Italy; lea.calo@unicatt.it (L.C.); jacopo.galli@unicatt.it (J.G.); 3 Complex Unit of Otolaryngology, Department of Aging, Neurological, Orthopedic and Head and Neck Sciences, Fondazione Policlinico Universitario A. Gemelli IRCCS, Largo A. Gemelli 8, 00168 Rome, Italy; 4 Department of Clinical Sciences and Translational Medicine, University of Rome Tor Vergata, Via Cracovia 50, 00133 Rome, Italy; alessandrociavarro@gmail.com

Periodic fever/aphthosis/pharyngitis/adenitis (PFAPA) syndrome was initially described in a small cohort of American children [1] who had early-onset and regularly recurrent febrile episodes and at least one of aphthous stomatitis, pharyngotonsillitis and cervical lymph node enlargement for whom upper respiratory tract infections and cyclic neutropenia had been ruled out [2]. Febrile attacks of PFAPA syndrome are usually encountered in children less than 5 years old, last from 2 to 5 days, and recur at a predictable frequency of about 3–6 weeks so that parents frequently note the fixed regularity of such fevers [3]. Laboratory tests performed during attacks show nonspecific neutrophil leukocytosis (which is useful for differentiating cyclic neutropenia) combined with significantly increased inflammatory markers, increased levels of proinflammatory cytokines in the serum, and the increased retention of activated T cells in many peripheral tissues [4], such as the tonsils, though no specific biomarker is yet available to confirm the diagnosis. All the described clinical characteristics of the attacks have good diagnostic sensitivity but low specificity; moreover, outside PFAPA attacks, the children grow harmoniously and have no symptoms of disease [5].

International groups attempted to generate new classification criteria for PFAPA syndrome to promote their inclusion in translational studies. Firstly, the recognition of this disorder requires the exclusion of the protean causes of recurrent fevers in children such as infectious, autoimmune, and malignant diseases or drug-induced reactions, and differential diagnosis should also consider hereditary periodic fever syndromes [6] caused by genetic defects of the innate immunity machinery, including familial Mediterranean fever, tumor-necrosis-factor-receptor-associated periodic syndrome, cryopyrin-associated periodic syndrome and mevalonate kinase deficiency [7,8,9,10]. Nevertheless, especially after the coronavirus disease 2019 pandemic, the PFAPA phenotype may harbor many unanswered questions, mostly in relation to issues of prevention, as the main burden for patients and their families is the exhausting process of excluding every potentially treatable cause of recurrent fevers, which is usually time-consuming [11,12,13].

There is no consensus on the best way to manage PFAPA patients, though many efforts to generate evidence-driven guidelines via the definition of standardized treatment plans have been made [14]. Unfortunately, no standard of care is available due to a lack of clinical trials and high-quality evidence-based data; indeed, there are no validated response measures, making comparisons between all potential treatments quite difficult. Febrile attacks in children respond well to low-dose betamethasone (0.1 mg/kg of body weight), which is highly effective in abating fever within a period from 2 to 6 h and controlling most disease symptoms, to the point that such a response, combined with a child’s well-being in the interfebrile periods, is considered crucial for diagnosis [15]. As a prophylactic strategy, cimetidine may be effective in up to 30% of patients [16], while colchicine has been tested with conflicting results regarding its overall effectiveness [17]. Tonsillectomy with or without adenoidectomy is a further option for patients with severely recurrent febrile attacks occurring every 7–10 days for a period of at least 6 months, and 20–80% of these cases (depending on the studies) may completely recover [18]. We should longitudinally investigate the management of PFAPA children and the impact of the disease on social issues, as objective measures of the quality of life in pediatric patients and their families are not available [19,20].

In general terms, the prognosis of PFAPA is favorable as most patients will outgrow their symptoms before adulthood [21]. Although PFAPA is commonly reported in children, different reports also claim evidence of it in adults [22,23]. A pathogenetic hypothesis to explain PFAPA attacks should involve inflammasomes, which mediate the production of interleukin (IL)-1β, and dysregulated IL-1β monocyte production was postulated in these patients [24]. Indeed, IL-1-related/interferon-induced genes were found to be significantly overexpressed during attacks [25], but the exact reason for the recurrence of the attacks remains undeciphered. In a recent study, Manthiram et al. suggested including PFAPA in the spectrum of Behçet’s disease based on the clinical similarities between the two conditions [26], and the demonstration of the activation of IL-1 during acute phases of PFAPA phases prompted the off-label use of IL-1 inhibitors in the most difficult-to-treat patients, which showed impressive results at both the clinical and laboratory levels [27].

Although PFAPA syndrome is a sporadic condition displaying some similarities with inflammasome-mediated autoinflammatory disorders [28], many studies have reported a familial clustering of the syndrome, suggesting a genetic basis with autosomal dominant transmission at reduced penetrance [29]. Because of the known sequelae of overlooking an autoinflammatory disorder, it is crucial to rule out pathogenic variants in autoinflammation-related genes via genetic testing for PFAPA cases. Indeed, polymorphisms in different genes involved in autoinflammatory kinetics such as *MEFV*, *NLRP3*, *TNFRSF1A*, *CARD8*, and *MVK* have been observed also in PFAPA patients.

In particular, a next-generation sequencing analysis which studied 32 genes involved in autoinflammation identified the frameshift variant C10X in the *CARD8* gene for 11 out of 82 unrelated PFAPA patients who were also shown to have a statistically significantly higher incidence of symptoms outside of attacks and an increased prevalence of oral aphthosis [30]. Furthermore, in a family with PFAPA syndrome, an exome analysis revealed missense variants in the *ALPK1* gene, which is usually active in the response to Gram-negative bacteria [31]. An increased prevalence of the *NLRP3* Q703K variant was also found in 21 patients with PFAPA syndrome, though with weak functional effects, as this polymorphism is present in almost 5% of the healthy population [32]. Therefore, no ”single” gene is currently associated with PFAPA syndrome, which should be regarded as a polygenic autoinflammatory disease or a complex disorder linked to Behçet’s disease and recurrent aphthous ulcers [33,34]. Most studies related to PFAPA patients hypothesize a multifactorial basis, while an environmental trigger should provoke inflammasome activation and periodically activate PFAPA attacks with fever and oral cavity/cervical manifestations [35].

A host of environmental triggers has been studied, but no definite culprit has been found for PFAPA syndrome. A potential hint to the disease might reside in the oral cavity, which hosts the second most abundant community of microbiota in the human body after the gut: bacteria, viruses, and fungi occupy different niches such as the teeth, gingival sulci, hard and soft palates, tongue, and tonsils, but the exact relationship between these districts and febrile PFAPA attacks remains unknown [36]. Variations in the microbiome’s composition and interactions among various taxonomic units have been associated with childhood recurrent upper airway infections, and the specific evaluation of the adenotonsillar lymphoid tissue microbiome has revealed the prevalence of proteobacteria in children with chronic tonsillitis as opposed to the prevalent fusobacteria and spirochetae in those with simple tonsillar hypertrophy and obstructive sleep apnea syndrome [37]. However, a definition of an infectious etiology remains elusive in PFAPA syndrome. The host’s dependence on the microbiome for both the establishment and maintenance of healthy status is demonstrated via comparisons of animals raised with and without a microbiome, in which the symbiosis of microbiota performed many beneficial functions for the host [38]. A histology evaluation of tonsil specimens from PFAPA children undergoing tonsillectomy identified chronic tonsillar inflammation with lymphoid hyperplasia and non-uniform immunological patterns [39]. Using next-generation sequencing technology, Tejesvi et al. compared the bacterial microbiota of tonsils removed from 30 PFAPA children with those of 24 non-PFAPA controls, finding a relative abundance of cyanobacteria and relative paucity of Streptococci in PFAPA cases [40].

A failure in the regulation of innate immunity cells, which are unable to recognize selected microbial communities of the tonsil ecosystem, prompting a febrile inflammatory response, might be the initiator of PFAPA syndrome: such a failure to identify components of the of oral microbiome for a few days in a month for different months might underpin the recurrence of fevers. The development of novel probiotics such as K12 from the oral-cavity-commensal species *Streptococcus salivarius* (SsK12) introduced the goal of specifically achieving oral health benefits from probiotics; this tool has been proven effective in reducing childhood upper respiratory tract infections, reducing children’s sick leave days or parents’ absence days from work, and reducing the number of days using antibiotics [41], but it may also restore innate immunity cells’ capability to recognize oral microbiota as commensals in PFAPA patients. In fact, the administration of SsK12 for at least 6 months was found to mitigate PFAPA syndrome in 85 children; in particular, SsK12 halved the total number of febrile attacks, shortened the duration of single attacks, and reduced the need for corticosteroids to abate fever [42].

In conclusion, PFAPA syndrome belongs to the family of autoinflammatory disorders, and its pathophysiology remains mysterious; it is probably related to oligo or polygenic mechanisms and recurs over a variable length of time in combination with clinical signs relating to the oral cavity and cervical lymph nodes. Pharyngitis and cervical adenitis are distinctive manifestations of PFAPA syndrome, which is largely underdiagnosed among the non-hereditary causes of pediatric fevers and is sometimes confused with other hereditary periodic fevers, primary or acquired immunodeficiency disorders, and cyclic neutropenia. Unfortunately, options for successfully managing the disease and stopping the recurrence of fevers are limited. Interventions focused on innate immunity cells might enhance their response and convey possible benefits for these children; how the microbiome interacts with peculiar genotypes to influence PFAPA expression is an open question, and the acquisition of metagenomic data will create opportunities to examine the evolutionary tuning of the microbiome to the host and shed light on the manifold human–microbiome partnership.
